# A Case of Dedifferentiated Melanoma With Lymph Node Metastasis Where Molecular Biological Tests Were Useful for Diagnosis

**DOI:** 10.7759/cureus.21644

**Published:** 2022-01-26

**Authors:** Minori Kamata, Takashi Minamisaka, Johji Imura, Katsuhiko Saitoh, Akiko Shimomura, Akira Noguchi

**Affiliations:** 1 Department of Diagnostic Pathology, Faculty of Medicine, Academic Assembly, University of Toyama, Toyama, JPN; 2 Department of Diagnostic Pathology, Toyama City Hospital, Toyama, JPN

**Keywords:** braf v600e mutation, immunohistochemistry, metastasis, dedifferentiation, melanoma

## Abstract

Malignant melanoma is known to have an altered phenotype and loss of differentiation markers for melanoma due to metastasis. Here, we report a case in which the expression of the immunohistochemical markers for melanoma was changed due to lymph node metastasis of primary cutaneous malignant melanoma. The patient, a male in his 60s, was diagnosed with malignant melanoma after undergoing excision of a skin mass. The additional excision specimen showed a small number of tumor cell clusters infiltrating the dermis. The biopsied lymph node showed completely different histological findings from those of the skin lesion and consisted of spindle-shaped tumor cells. An immunohistochemical study revealed no significant positive reactions in the lymph node tissue indicative of melanoma. The additional genetic study revealed BRAF V600e mutations in both the primary tumor and a lymph node. Together with the histological findings, the diagnosis was of metastasis of dedifferentiated melanoma to a lymph node. In summary, there is a risk of underestimation or misdiagnosis of melanoma as undifferentiated sarcoma or other tumors when melanoma metastasizes to lymph nodes and findings show a dedifferentiated or undifferentiated tumor. Therefore, as in this case, it is necessary to add a genetic study in order to make a comprehensive judgment.

## Introduction

Malignant melanoma is a common and highly malignant cutaneous neoplasm. Diagnosis of this tumor is based on topographical histological findings, and often on an immunohistochemical examination as an adjunct [1]. Melanoma is characterized by the presence of melanin pigment in its cytoplasm, but occasionally amelanotic melanoma without melanin pigment production is encountered. There are also various histological subtypes of melanoma [2]. In addition, there are melanoma subtypes that show different differentiation; as a result, the morphology may differ significantly [3]. Thus, immunohistochemical examination is useful for differential diagnosis of melanoma.

Melan-A and HMB45 are well-known markers for melanoma, but both are expressed in response to the development of melanosomes in tumor cells [4] and are often negative in dedifferentiated or undifferentiated melanoma [5]. S100 is a typical melanoma marker, but although it is susceptible, its specificity is low, as it is positive in other neural tumors [6]. Recently, SOX10 has become known as the most sensitive marker. These latter two, however, have limited specificity compared to the former two [7]. On the other hand, it is also known that the phenotypes of primary and metastatic melanoma can differ significantly [1].

This study reports a case of cutaneous melanoma metastasized to lymph nodes. It was finally diagnosed as dedifferentiated melanoma after an additional genetic study since the histological findings and immunophenotype were of large differences between the two lesions and conventional tools had difficulty in making a differential diagnosis.

## Case presentation

The patient, a male in his 60s, had been aware of a mass on the occipital skin for 10 years. Because of easy bleeding, he had recently been excised at another hospital and histopathologically diagnosed with malignant melanoma in p-T4aNXMX. An additional skin resection and sentinel lymph node biopsy were subsequently performed.

Pathological findings

**Figure 1:**
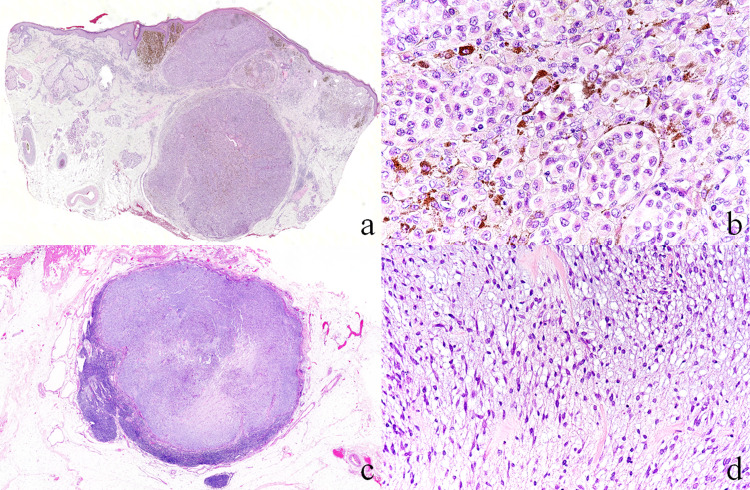
Histological findings in hematoxylin-eosin staining - (a, b) a primary lesion and (c, d) a lymph node metastatic lesion. (a) Well-defined tumor nodules were present mainly in the dermis (×5). (b) Tumor nests are composed of typical melanoma cells with melanin pigments (×40). (c) Lymph node is mostly replaced by well-defined metastatic lesions (×8). (d) Metastatic lesion is composed of spindle tumor cells (x20).

The initial skin resected material showed no ulceration and a relatively well-defined solid nodular lesion of 7 mm in longitudinal diameter present within the dermis. There were only a few intraepidermal lesions, and most of the intradermal lesion was predominant. The border between the epidermis and the intradermal lesion was clear, and the epidermis was thinned by tumor tissue compression (Figure 1, panel a). The tumor cells constituting the nodules had round nuclei with well-defined nucleoli. The cytoplasm was broad and weakly eosinophilic, with a fine-grained appearance. Tumor cells containing abundant light brownish melanin pigment were found in regional clusters, while unicellular cells with pigment granules were present sporadically in the nodules mainly composed of cells lacking melanin (Figure 1, panel b). Based on these findings, the patient was pathologically diagnosed with nodular malignant melanoma.

The Breslow tumor thickness was 5 mm, and the Clark level was IV. There was no obvious lymphatic or vascular invasion or perineural invasion. There were no tumor cells exposed at the resection margin, which was 25 μm at the shortest. Additional excised skin tissue showed a small cluster of tumor cells that had invaded the dermis. The sentinel lymph node formed a well-defined nodule encapsulated with thin, membranous fibrous tissue, and the existing lymph node tissue was partly remained (Figure 1, panel c). Within the nodule, tumor cells were seen, arranged in a bundle with myxomatous stroma. They were mainly spindle-shaped, but with some round cells. The nuclei of the tumor cells were spindle-shaped to oval, and some cells had a slightly irregular nuclear shape. The cytoplasm was delicate or indistinct without the cellular process (Figure 1, panel d). There was no unique arrangement, or cellular cluster-like epithelial tumors, or other morphological features, such as a giant rhabdoid cell. Based on these morphological findings, a spindle cell tumor was suspected.

Immunohistochemical findings

**Figure 2:**
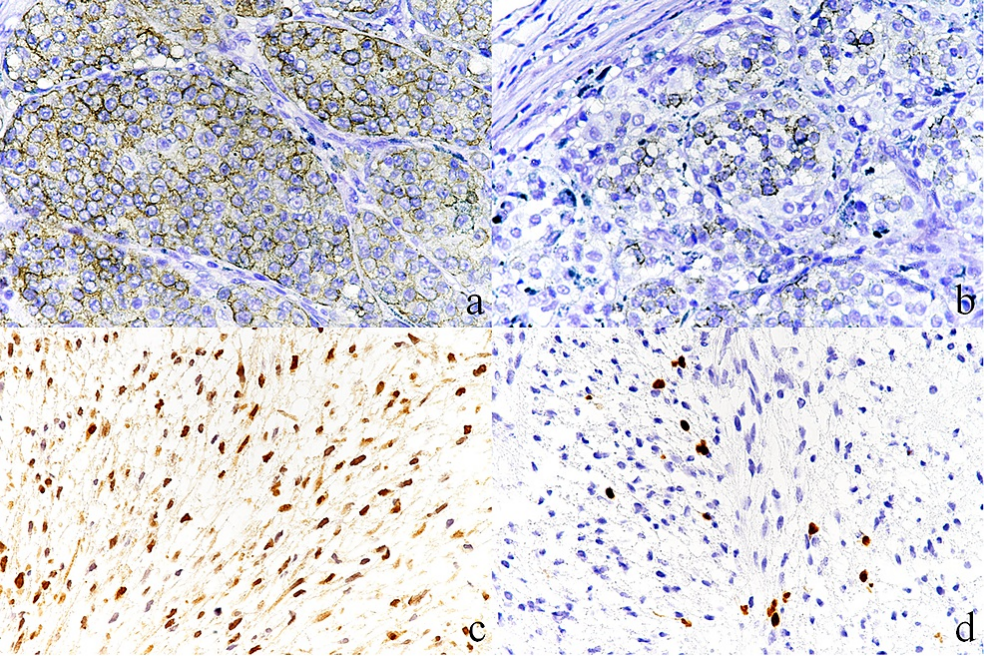
Immunohistochemical findings - (a, b) primary lesions and (c, d) metastatic lesions. (a) Cytoplasmic granular reactions are noted for Melan-A. (b) A small number of tumor cells is a positive reaction for HMB45. (c) Positive reaction for S100 is observed in the nucleus of a spindle tumor cell. (d) SOX10 positive nuclear reactions are seen in the isolated spindle cells (a-d, ×30).

Cytokeratin as an epithelial marker was positive for CAM5.2 diffusely only in lymph nodes. The melanoma markers Melan-A and HMB45 were positive in the cytoplasm of tumor cells locally in the primary tumor but negative in the spindle-shaped cells of lymph nodes (Figure 2, panels a and b). S100 was positive in both lesions (Figure 2, panel c). SOX10 was positive only in the nucleus of single isolated cells in a lymph node lesion (Figure 2, panel d). In addition, vimentin was diffusely positive in both lesions.

Genetic findings

**Figure 3:**
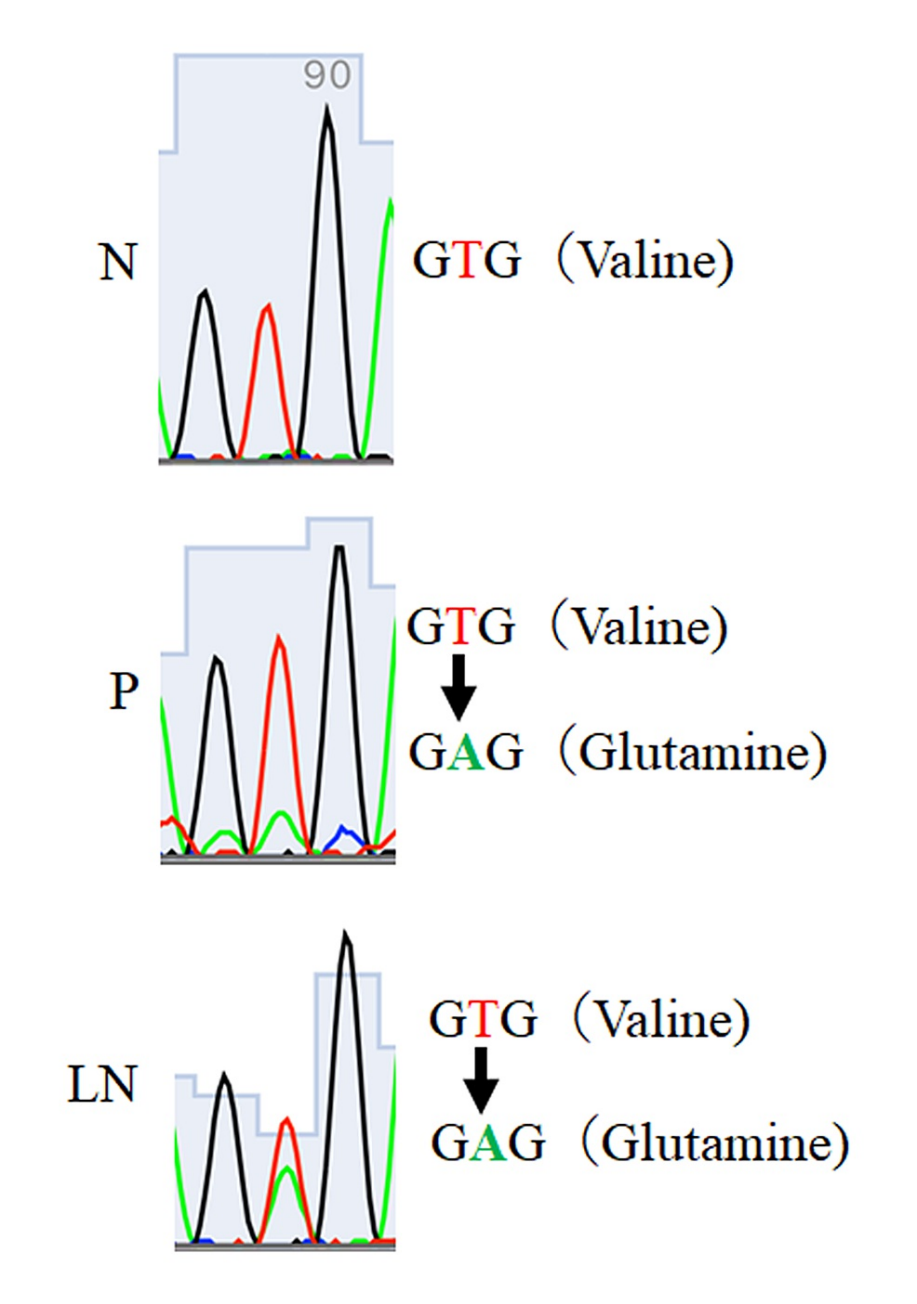
Results of Sanger sequencing for BRAF exon 15. The same T to A base substitution, amino acid replacement from valine to glycine, is shown in P and LN. The V600E mutation occurs in both lesions. N: normal skin; P: primary lesion; LN: lymph node metastatic lesion

Polymerase chain reaction (PCR) amplifications were performed for detection of BRAF exon 15 and NRAS exon 2 and 3 mutation, using Sanger sequencing with gDNA extracted from paraffin sections of the primary tumor and lymph nodes. In both primary and lymph node metastases, a common mutation in BRAF V600e was found, an amino acid substitution from valine to glutamine with a T to A rearrangement (Figure 3) [8]. In addition, NRAS was a wild type in both lesions.

Postoperative period

The patient was considered to be a case of in-transit metastasis, due to the presence of clustering tumor cells in the additional resection specimen as a satellite lesion and lymph node metastasis. Since the patient also had BRAF mutation, combination therapy with dabrafenib as BRAF inhibitor and trametinib as MEK inhibitor is currently being administered. No other lesions have been found.

## Discussion

It is common for melanoma to change phenotype when metastasizing or recurring. In such cases, immunohistochemical examination is necessary for differential diagnosis, and aberrant expression of unexpectedly positive melanoma markers may be encountered [9]. On the other hand, so-called heterologous differentiation, which is the loss of melanoma characteristics due to dedifferentiated or undifferentiated transformation, has been observed [5]. Among these, dedifferentiated melanoma often has a so-called sarcoma-like appearance [10-12]. It may also contain an adenocarcinoma component when it includes glandular duct formation and is recognized as a collision tumor [13]. Among the former, it may be misidentified as spindle cell sarcoma or undifferentiated sarcoma such as undifferentiated pleomorphic sarcoma (UPS) [5,14]. The spindle cell melanoma is misdiagnosed as schwannoma and sometimes as intranodal schwannoma, even if in a lymph node [15], and can easily be viewed as a false-negative in sentinel lymph node diagnosis [5].

In recent years, immunohistochemical examination has been increasingly used as an adjunct to morphological diagnosis, but there is a risk of misdiagnosis through overconfidence. In melanoma, Melan-A and HMB45 are highly specific markers, but a negative result does not rule out melanoma. Both markers are expressed during melanocyte differentiation, and undifferentiated melanoma may not have well-developed melanocytes, which may reduce the frequency of positive results [4,5]. SOX10 is also known to be a sensitive marker in melanoma [7], but in the present case, skin lesions were negative and only a few isolated cells in lymph node lesions were positive. Careful observation is required, without overlooking positive cells. On the other hand, because SOX10 is a pan-schwannian marker, it may not be suitable for differentiating melanoma from schwannoma, and it may be important to look for morphological features such as cell morphology and unique arrangements such as Antoni A and B types [7]. Thus, it should be noted that immunohistochemical examination has many limitations in the diagnosis of melanoma.

Thus, in order to solve the problem of classical histopathological findings or immunohistochemical examination, BRAF V600e mutation search may become a surrogate marker for melanoma diagnosis. Certainly, Melan-A and HMB45 have been used as specific markers for conventional melanoma. But in some cases, including our case, their immunohistochemical marker is negative and must be supplemented with an additional method. From this perspective, BRAF V600e mutation is also a characteristic mutation of melanoma and can be a useful marker for differential diagnosis. The genetic study could be applied to the actual diagnosis of melanoma, such as in our case. For example, it could help differentiate soft tissue sarcomas with UPS-like histological findings that have occurred in patients with a history of melanoma. In fact, none of these soft tissue tumor cases was found to have BRAF V600e mutations [14].

Other extensive studies have also found no BRAF V600e mutations in sarcomas [16,17]. These reports also indicate that BRAF V600e mutations may not act oncogenically in sarcoma development. However, it should be noted that there is a report of BRAF V600e mutations in four schwannomas and one malignant peripheral nerve sheath tumor case [18]. Thus, BRAF V600e and NRAS mutations in melanoma are considered to be as specific as in lung cancer. In fact, melanoma is one of the few tumors where treatment options are limited, and BRAF V600e inhibitors are currently used as molecular targeted therapies.

This patient was also found to have a BRAF V600e mutation and is being treated with a combination of BRAF and MEK inhibitors. In a previous report, BRAF V600e mutations in dedifferentiated cases occurred in 36% of patients with prior melanoma, and NRAS mutations were similar [5]. This was slightly lower than the frequency of mutations in conventional melanoma [19,20]. Therefore, it is unlikely that BRAF V600e mutations revert to the wild type in melanoma cells without BRAF V600e mutations upon dedifferentiation from the primary tumor. It may be that only non-BRAF V600e mutated cells subclone and proliferate from undifferentiated cells that arise in the heterogenous differentiation of the primary tumor. Further studies to substantiate this hypothesis are expected.

## Conclusions

It is important to keep in mind that the morphological and immunohistochemical characteristics of ordinary melanoma may change as the tumor cells dedifferentiate, as in this case. Great care must be taken to avoid misjudgment in the diagnosis of metastatic lesions. For this reason, we would like to emphasize the necessity of differential diagnosis using molecular biological techniques as in this case.
